# Music-Enhanced Analgesia and Antiseizure Activities in Animal Models of Pain and Epilepsy: Toward Preclinical Studies Supporting Development of Digital Therapeutics and Their Combinations With Pharmaceutical Drugs

**DOI:** 10.3389/fneur.2019.00277

**Published:** 2019-03-27

**Authors:** Cameron S. Metcalf, Merodean Huntsman, Gerry Garcia, Adam K. Kochanski, Michael Chikinda, Eugene Watanabe, Tristan Underwood, Fabiola Vanegas, Misty D. Smith, H. Steve White, Grzegorz Bulaj

**Affiliations:** ^1^Department of Pharmacology and Toxicology, University of Utah, Salt Lake, UT, United States; ^2^Department of Medicinal Chemistry, University of Utah, Salt Lake, UT, United States; ^3^Greatful Living Productions, Salt Lake, UT, United States; ^4^Department of Atmospheric Sciences, University of Utah, Salt Lake, UT, United States; ^5^The Gifted Music School, Salt Lake, UT, United States; ^6^The School of Music, University of Utah, Salt Lake, UT, United States; ^7^The School of Dentistry, University of Utah, Salt Lake, UT, United States; ^8^School of Pharmacy, University of Washington, Seattle, WA, United States

**Keywords:** neuropathic pain, cancer pain, arthritis, opioids, inflammation, refractory epilepsy, epileptic seizures, mobile medical apps

## Abstract

Digital therapeutics (software as a medical device) and mobile health (mHealth) technologies offer a means to deliver behavioral, psychosocial, disease self-management and music-based interventions to improve therapy outcomes for chronic diseases, including pain and epilepsy. To explore new translational opportunities in developing digital therapeutics for neurological disorders, and their integration with pharmacotherapies, we examined analgesic and antiseizure effects of specific musical compositions in mouse models of pain and epilepsy. The music playlist was created based on the modular progression of Mozart compositions for which reduction of seizures and epileptiform discharges were previously reported in people with epilepsy. Our results indicated that music-treated mice exhibited significant analgesia and reduction of paw edema in the carrageenan model of inflammatory pain. Among analgesic drugs tested (ibuprofen, cannabidiol (CBD), levetiracetam, and the galanin analog NAX 5055), music intervention significantly decreased paw withdrawal latency difference in ibuprofen-treated mice and reduced paw edema in combination with CBD or NAX 5055. To the best of our knowledge, this is the first animal study on music-enhanced antinociceptive activity of analgesic drugs. In the plantar incision model of surgical pain, music-pretreated mice had significant reduction of mechanical allodynia. In the corneal kindling model of epilepsy, the cumulative seizure burden following kindling acquisition was lower in animals exposed to music. The music-treated group also exhibited significantly improved survival, warranting further research on music interventions for preventing Sudden Unexpected Death in Epilepsy (SUDEP). We propose a working model of how musical elements such as rhythm, sequences, phrases and punctuation found in K.448 and K.545 may exert responses via parasympathetic nervous system and the hypothalamic-pituitary-adrenal (HPA) axis. Based on our findings, we discuss: (1) how enriched environment (EE) can serve as a preclinical surrogate for testing combinations of non-pharmacological modalities and drugs for the treatment of pain and other chronic diseases, and (2) a new paradigm for preclinical and clinical development of therapies leading to drug-device combination products for neurological disorders, depression and cancer. In summary, our present results encourage translational research on integrating non-pharmacological and pharmacological interventions for pain and epilepsy using digital therapeutics.

## Introduction

Pain and epilepsy are distinct neurological conditions which share unmet needs for innovating treatments. People living with chronic pain have limited options for pain relief. Non-steroidal anti-inflammatory drugs (NSAIDs) are commonly used to treat chronic pain, despite their gastrointestinal and cardiovascular toxicities. Opioid-based pain management has considerable adverse effects, in addition to abuse potential [e.g., the opioid epidemic in the US resulted in over 47,000 deaths in 2017 due to overdosing opioids (1)]. People with epilepsy also face multiple challenges including: (1) seizure control (estimated 30% are refractory to current antiseizure drugs), (2) medication non-adherence, tolerability and adverse effects related to antiseizure drugs, (3) significantly higher mortality, and (4) co-morbidities requiring additional therapies. Taken together, novel approaches to chronic pain management and control of epileptic seizures will benefit millions of people living with epilepsy and pain worldwide (2, 3).

To innovate treatments for neurological disorders, we have been studying diverse strategies to integrate digital health technologies with CNS drugs (4–6). Digital health is a branch of healthcare that employs internet, digital, and mobile technologies for improving health and/or treating diseases. Non-pharmacological modalities such as behavioral therapies, music and disease self-management can be delivered by mobile health (mHealth) apps (focused on self-managements and well-being), while digital therapeutics (mobile medical apps) are intended to treat specific medical conditions. Several mobile apps and video games (Table S1) received approval, or clearance, from the Food and Drug Administration (FDA) using software as a medical device (SaMD) regulatory mechanism (7, 8). There are also increased interests in developing mobile apps for people living with chronic pain (9–16) and epilepsy (6, 17–19).

Since digital technologies can deliver music-based interventions (20), these opportunities are relevant for treatments of pain, epilepsy, stroke, dementia, and other chronic medical conditions (21–27). Music showed clinical benefits of reducing acute and chronic pain (25–27). For epilepsy, there is clinical and preclinical evidence that specific musical compositions exert anticonvulsant effects (4, 28–32). Clinical studies showed that exposure to Mozart's K.448 sonata in pediatric epilepsy patients, even those with refractory seizures, caused a significant reduction of seizure frequency and epileptiform discharges (33–39). These antiseizure effects were also observed in patients with their first unprovoked seizure (40), in those with drug-refractory epileptic encephalopathies (41), and in adult patients with epilepsy (42). In addition, the antiseizure effects of Mozart's K.448 have been confirmed in a rat model of acquired epilepsy (34). From a translational research perspective, there is a gap between clinical and preclinical studies on music-based interventions with only few reports describing effects of music in animal models of epilepsy (34), cancer pain (43), or affective disorders (44).

Preclinical studies on non-pharmacological interventions include environmental enrichment (EE) and exposure to music. EE comprises animal housing conditions which provide physical exercise, cognitive stimulation, and also favor social interactions (45, 46). EE can exert such effects as reduction and prevention of epileptic seizures, as well as analgesic effects in various neuropathic pain models in rats and mice (45, 47–51). Similarly to EE, music produces diverse physiological responses in rodents including improved memory, analgesia, antidepressant and antiseizure activities, as illustrated in Table 1. Music-based interventions in rodents affect neuroplasticity, neurochemical changes, immune responses, and the parasympathetic nervous system (59). Positive effects of EE and music suggest that preclinical studies on these non-pharmacological modalities can lead to novel combination therapies with pharmacological treatments (60, 61). In this work, we explored this repositioning (repurposing) strategy by testing how antiseizure musical compositions can also exert analgesic effects. Our present study of music-based intervention in mouse models of epilepsy and pain also serves as a preclinical surrogate for development of music-based digital therapeutics and their combinations with neuropharmacological therapies.

**Table 1:** Examples of music-based interventions studied in rodents.

**Species**	**Main outcomes**	**References**
Mouse	Modification of expression of multiple genes and improved memory	(52)
Mouse	Decreased anxiety in some genotypes and increased TrkB in all genotypes	(44)
Mouse	Antidepressant activity in combination with enriched environment housing	(53)
Rats	Higher pain thresholds and smaller tumors in bone cancer model	(43)
Rat	Significantly improved spatial memory impairment caused by status epilepticus	(54)
Rat	Decreased seizure frequency and number of high-frequency EEG spikes	(34)
Rat	Lowered heart rate in hypertensive animals	(55)
Rat	Modulation of BDNF expression in the hippocampus and hypothalamus	(56, 57)
Rat	Reduced metastatic nodules in animals injected with carcinosarcoma cells	(58)

## Methods

### Animals

Adult male CF-1, CD-1, and C57Bl/6 mice (Charles River, Kingston, NY, United States) or adult male Sprague-Dawley rats (Charles River, Raleigh, NC, United States) were used in specific experiments, as described below. Animals were housed in a temperature-, humidity-, and light-controlled (12 h light:dark cycle) facility. Animals were group housed and permitted free access to food and water. All experimental procedures were performed in accordance with the guidelines established by the National Institutes of Health (NIH) and approved by the University of Utah's Animal Care and Use Committee (IACUC).

### Music Intervention

The playlist used in the experiments comprised the Mozart compositions previously studied in clinical trials in people with epilepsy (4, 8–14, 16, 22). All compositions were in Major keys and included concertos, sonatas, and symphonies featuring varied types of instrumentation. The playlist was organized with a symphonic-based structure made up of two faster-paced “allegro” sections separated by a slower “adagio” section. The order of compositions was selected to balance arousal and optimize transitions between individual musical pieces. The first movement of the K.448 has been the most frequently studied of all the pieces, and was therefore featured multiple times throughout the playlist including the beginning and end of the first and third sections, as well as in the middle of the first section where it would normally be placed due to key (D major).

Each musical piece, with the exception of K.448, was featured once and arranged by key within the movement corresponding to its tempo. This arrangement corresponded to the “Circle of Fifths,” which is also commonly used in the modulatory progressions of Mozart and his contemporaries. The pieces were arranged in order of increasing number of sharps and decreasing number of flats in the key signature. The rationale for this order was to minimize any jarring transitions, with the intent to optimize entrainment and minimize any potential stress on the mice. The total playtime for the list was 3 h 4 min. The playlist was repeated three times separated by 1-h silence over 12-h dark-cycle at the average loudness of 64 dB with 71 dB peaks. The daily exposure to music was similar to that reported previously (19), except that Xing and colleagues used only K.448 in their experiments. The final order of musical compositions delivered in three parts was: Part 1: Sonata for Two Pianos in D Major, K.448: I. Allegro con spirit; Symphony No. 41 in C Major K.551 Jupiter III; Symphony No. 41 in C Major K.551 Jupiter IV; Piano Sonata No. 15 in C Major Sonata Semplice K545 III; Violin Concerto No.4 in D Major, K.218: I. Allegro; Sonata for Two Pianos in D Major, K.448: I. Allegro con spirit; Flute Concerto No.2 in D Major, K.314: I. Allegro aperto; Piano Concerto No.22 in E Flat, K.482: 1. Allegro; Piano Concerto No.22 in E Flat, K.482: 3. Allegro; Violin Concerto No. 1 in B Flat K.207 I; Violin Concerto No. 1 in B Flat K.207 III; Sonata for Two Pianos in D Major, K.448: I. Allegro con spiri. Part 2. Symphony No. 41 in C Major K.551 Jupiter II; Piano Sonata No. 15 in C Major Sonata Semplice K.545 II; Sonata for Two Pianos in D Major, K.448: II. Andante; Piano Concerto No.22 in E Flat, K.482: 2. Andante; Violin Concerto No. 1 in B Flat K.207 II Adagio. Part 3. Sonata for Two Pianos in D Major, K.448: I. Allegro con spirit; Mozart: Symphony #41 In C, K.551, “Jupiter”-3. Menuet & Trio; Piano Sonata No. 15 in C Major Sonata Semplice K.545 I; Symphony No. 46 in C K.96 I; Symphony No. 41 in C Major K.551 Jupiter I; Symphony No. 41 'Jupiter', K.551: 4th movement; Sonata for Two Pianos in D Major, K.448: I. Allegro con spirit; Sonata for Two Pianos in D Major, K.448: III. Molto allegro.

Music-exposed animals were kept in a separate room during the dark cycle in order to undergo music exposure, and were moved to normal housing facilities during the light cycle. Kindling animals were exposed to music beginning at the onset of kindling. All animals were exposed to music for a minimum of 3 weeks before testing. In order to assure music delivery at the proper volume, prior to the experiments the volume has been adjusted so that the average sound level during the playlist was 70 dB. The measurements, performed using the dB Meter iPhone app, were taken in the cages with fixed location of the speakers (Bose Companion20). The volume was optimized and the playlist delivered using a Mac laptop computer. The automation of music delivery was facilitated using the Apple Automator script. This program was responsible for setting the volume at the predetermined level and playing the music each day at the exact same time. The script was also set up to send out e-mail notifications each time when the music was played so that in case of a computer failure the lab personnel could start the playlist manually. Additionally, the Apple Remote Desktop was configured to enable remote computer control without disturbing the experimental environment. Mice in the control group were exposed to normal ambient noise continually. To control for daily transferring between music exposure rooms and standard housing rooms, control mice were transferred between testing rooms once daily.

### Drug Preparation

All test compounds were administered to mice in a volume of 10 ml/kg. *Ibuprofen*. Ibuprofen was suspended in 0.5% methylcellulose (Sigma-Aldrich, St. Louis, MO) and vortexed prior to administration to ensure complete dissolution or micronized suspension. It was concentrated to 2.5 mg/ml and given at a dose of 25 mg/kg body weight by intraperitoneal (i.p.) injection. Testing occurred 30 min following treatment. *Levetiracetam (LEV)*. LEV was suspended in 0.5% methylcellulose (Sigma-Aldrich, St. Louis, MO) and vortexed at least 15 min prior to administration to ensure complete dissolution or a micronized suspension. It was concentrated at a 40 mg/ml, and given at a dose of 400 mg/kg body weight. Testing occurred 1 h after i.p. injection. *CBD*. CBD was suspended in a suspension of 1:1:18 (ethanol, cremaphor, and phosphate-buffered saline, PBS) by initial dissolution in ethanol followed by addition of cremaphor and slow (drop-wise) addition of PBS. Each step included vigorous mixing (by vortex) for several minutes to ensure dissolution. The final test solution was prepared at a concentration of 10 mg/ml and was administered at a dose of 100 mg/kg. Testing occurred 2 h after injection. *NAX 505-5*. NAX 505-5 was suspended in a 1.0% (w/v) Tween 20 solution (prepared in PBS) and mixed gently prior to administration to ensure complete dissolution or a micronized suspension. It was prepared at a concentration of 0.4 mg/ml, and administered at a dose of 4 mg/kg. Testing occurred 1 h after i.p. injection.

### Carrageenan Assay

Localized inflammation in the hindpaw of mice was induced by injecting carrageenan (25 μl, 2% in 0.9% NaCl, λ-carrageenan; Sigma-Aldrich, St. Louis, MO) subcutaneously into the plantar surface of the right (ipsilateral) hindpaw, as previously described (62). Carrageenan-induced inflammation was verified (3 h post-carrageenan) using a caliper to assess paw edema across the dorso-ventral aspect of both the carrageenan-injected (ipsilateral) and the non-injected (left paw, contralateral) hindpaw. Paw withdrawal responses from thermal stimulation were assessed according to previously described methods (63–65). Approximately 2.5 h after carrageenan injection, mice were placed in plexiglass chambers on top of a heated glass surface (30°C) (IITC, Woodland Hills, CA, United States). Testing coincided with the time of peak hyperalgesia following carrageenan, as previously described (62, 66), 3 h following carrageenan. Thermal stimulation was applied with a projection bulb (IITC, Woodland Hills, CA, United States; 53 mJ, 35% of maximal stimulus intensity) below the heated glass surface. Latency to paw withdrawal was measured from the onset of thermal stimulation until a full paw withdrawal occurred. Three measurements were obtained from each paw, with at least 1 min between assessments, and subsequently averaged to obtain the mean paw withdrawal latency. Because of the ease of testing and positive, reproducible data in music vs. control studies in both CF-1 and CD-1 strains, this model was chosen to determine drug efficacy in combination with music in the CD-1 strain. In drug treated groups, LEV, CBD, or 505-5 were administered by i.p. injection, 2 h following carrageenan paw injection and IBU was administered by subcutaneous injection 2.5 h following carrageenan paw injection such that the time-to-peak effect (TPE) would match the time of peak inflammation following carrageenan (1 h post LEV, CBD, 505-5 or 30 min post IBU; 3 h post-carrageenan). Separate groups of mice were also treated with Veh, and Veh-treated mice were pooled (*N* = 8) comprising individual groups of Veh-treated mice (*N* = 2) that were treated alongside each drug group.

### Plantar Incision Assay

The CD-1 mouse plantar incision assay was performed in a similar manner to previously described methods (66). Mice were anesthetized using 0.4–0.5% isoflurane and received a 5 mm incision on the plantar surface of the left hindpaw as well as separation and elevation of the underlying plantaris muscle. The muscle was replaced and the wound sutured. We previously determined that mechanical allodynia following plantar incision were comparable both 1 and 2 days following the initial injury but that responses were more consistent on the second day (data not shown). Hyperthermia latency was comparable from 4 h to 2 days after surgery (data not shown), and so was tested the afternoon after morning surgery. *Hyperthermia*. Test was performed on the day of surgery, allowing at least 4 h of recovery time. Paw withdrawal responses from thermal stimulation were assessed according to methods described above for thermal testing in the carrageenan model. *Von Frey Filaments*. Two days following surgery, mice were placed on an elevated wire mesh rack (~24 inches high) in plexiglass cages. This allowed for access of the plantar surface of the hindpaw and testing using von-Frey monofilaments. Filaments (10 g maximum fiber stimulation) were applied to the mid paw plantar surface near the incision. Individual paw responses to mechanical stimulation were quantified using the Dixon up-down method and allowed for determination of the 50% paw withdrawal threshold (PWT).

### Corneal Kindling Assay

CF-1 mice were kindled according to the optimized protocol defined by Matagne and Klitgaard (67) and Rowley and White (68). Briefly, mice were stimulated twice daily (5 days/week) with a corneal stimulation of 3 mA (60 Hz) for 3 s. Prior to each stimulation, a drop of 0.9% saline containing 0.5% tetracaine hydrochloride (Sigma-Aldrich, St. Louis, MO, United States) was applied to the cornea to ensure local anesthesia and good electrical conductivity. Stimulations were delivered 4 h apart. Animals were considered kindled when they displayed five consecutive stage five seizures according to the Racine scale (69, 70).

### Viral Infection-Induced Seizure Assay

Mice (C57Bl/6J; The Jackson Laboratory) exposed to the music intervention for 21 days, or to the standard housing conditions (control), were infected with Theiler's Murine Encephalomyelitis Virus (TMEV) by intracortical administration as described elsewhere (71, 72). During the 7-day post viral infection period, the animals were evaluated for acute handling-induced seizure severity.

### Statistical Analysis

Single group comparisons were made using a *t*-test. Multiple group comparisons were made using a one-way or a two-way ANOVA followed by a Newman-Keuls or a Bonferroni *post-hoc* test. Data analysis were completed using statistical software (GraphPad Prism). A *p* < 0.05 was considered significant. Data are presented as means ± standard error. Animals protected (without seizure) were compared to those with seizures by the Fisher's exact test (corneal kindling). Survival in the kindling model was evaluated using the Log-rank (Mantel-Cox) and Gehan-Breslow-Wilcoxon tests. In the TMEV model, behavioral seizures were evaluated using the Mann-Whitney *U*-test.

## Results

In order to test effects of music on analgesia and antiseizure activity in animal models of epilepsy and pain, we created a playlist comprising Mozart compositions previously shown to reduce epileptic seizures or epileptiform discharges in PWE (28, 31, 35, 36, 41, 42, 73, 74). The playlist was prepared and delivered as described in the Methods section. Mice were either exposed daily to music for at least 21 consecutive days (the music-treated group), or maintained under ambient noise in the standard housing conditions (the control group).

### Analgesic Assays

**Figure 1:**
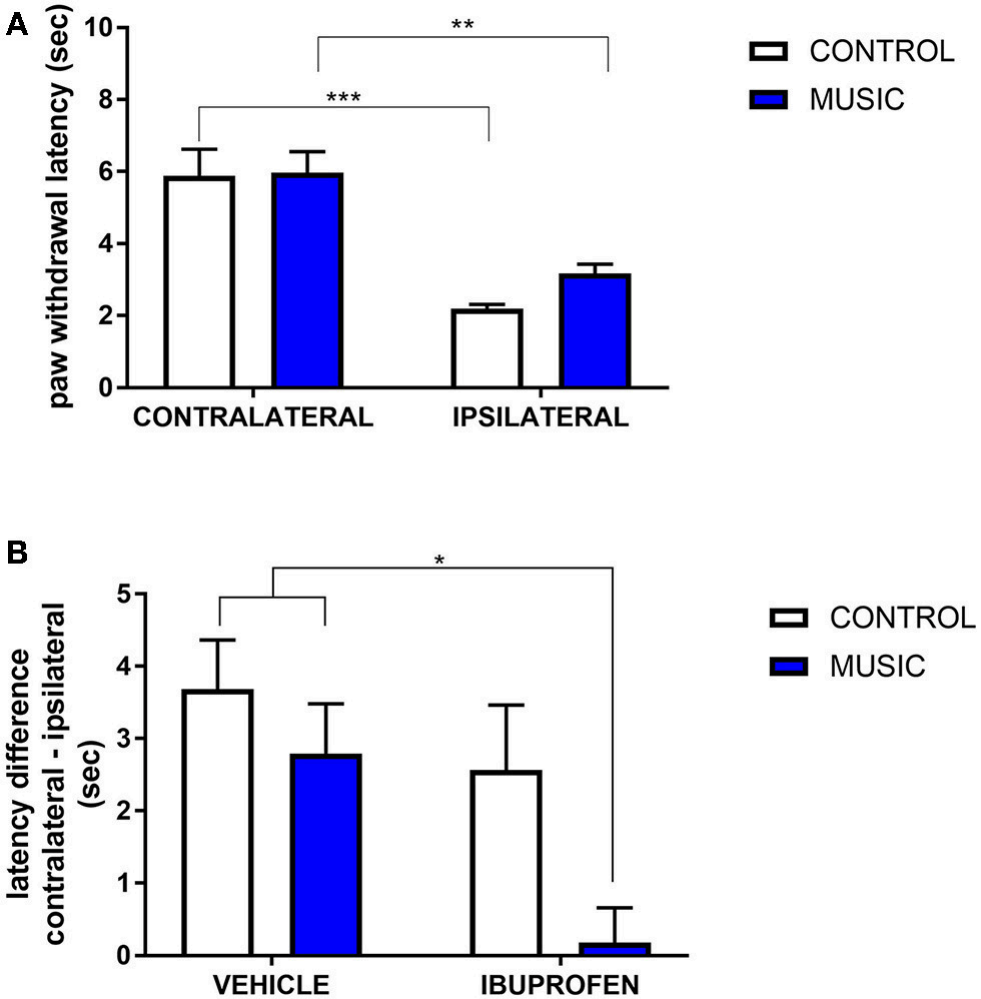
Analgesic effects of music-based intervention in the carrageenan model of inflammatory pain in CD-1 mice. **(A)**—effects of 21-day exposure to the Mozart playlist (MUSIC–blue; CONTROL–white) on paw withdrawal latency. Carrageenan-injected (ipsilateral paws) show thermal hyperalgesia (reduced withdrawal latency) in comparison to non-injected (contralateral) paws. **(B)**—effects of music (MUSIC–blue; CONTROL–white) on activity of ibuprofen following i.p. administration. Ibuprofen was administered at a dose of 25 mg/kg, 30 min prior to testing. Hyperalgesia was observed in animals exposed to control conditions whereas music-exposed and ibuprofen-treated animals show a normalized response to thermal stimulation (reduction of hyperalgesia). *N* = 5–8 per group. ^*^P < 0.05, ^**^P < 0.01, ^***^P < 0.001. Data were analyzed using a two-way ANOVA followed by a Bonferroni *post-hoc* test.

**Figure 2:**
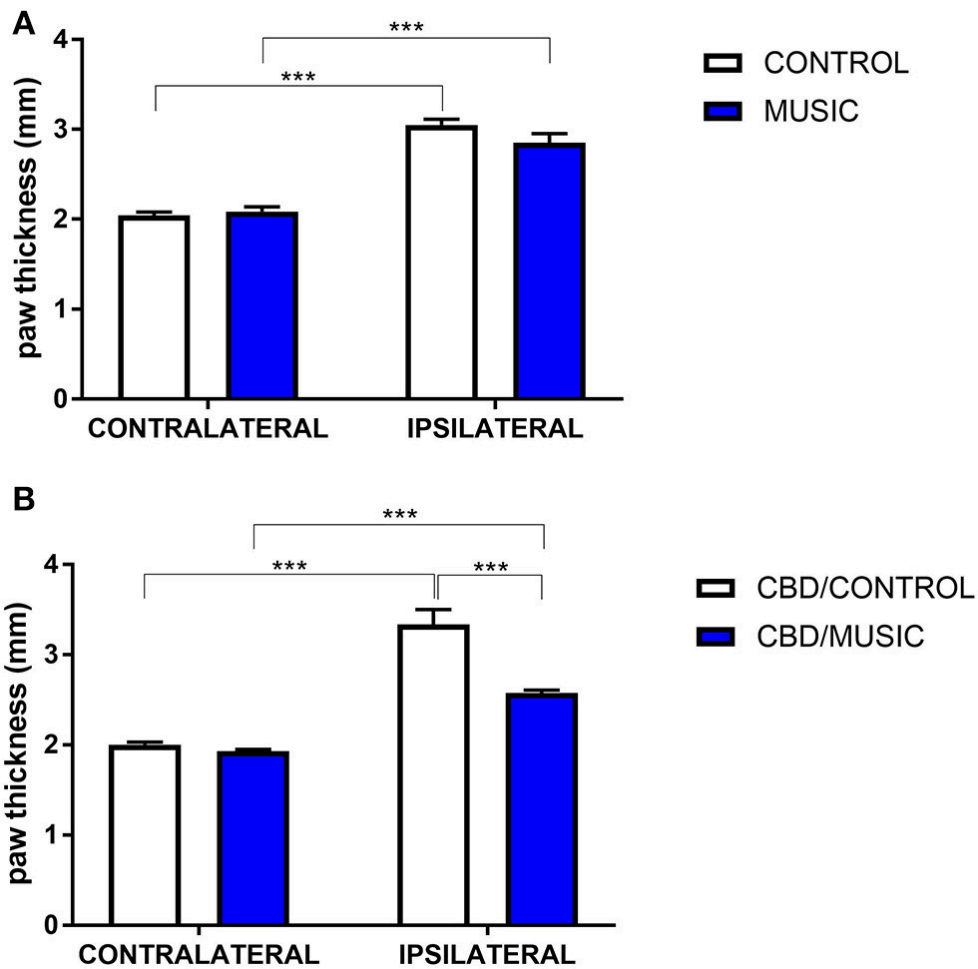
Effects of music-based intervention on paw thickness (edema) following carrageenan administration in CD-1 mice. **(A)**—Carrageenan-injected (ipsilateral paws) show increased thickness (caliper measurement across the dorso-ventral aspect of the paw) compared to non-injected (contralateral) paws (MUSIC–blue; CONTROL–white). **(B)**—effects of music (MUSIC–blue; CONTROL–white) on activity of Cannabidiol (CBD, 100 mg/kg) following i.p. administration. CBD was administered 120 min prior to testing. Edema was observed following carrageenan in animals exposed to control conditions whereas music-exposed and CBD-treated animals show a diminished edema. *N* = 6–8 per group. ^***^P < 0.001. Data were analyzed using a two-way ANOVA followed by a Bonferroni *post-hoc* test.

**Figure 3:**
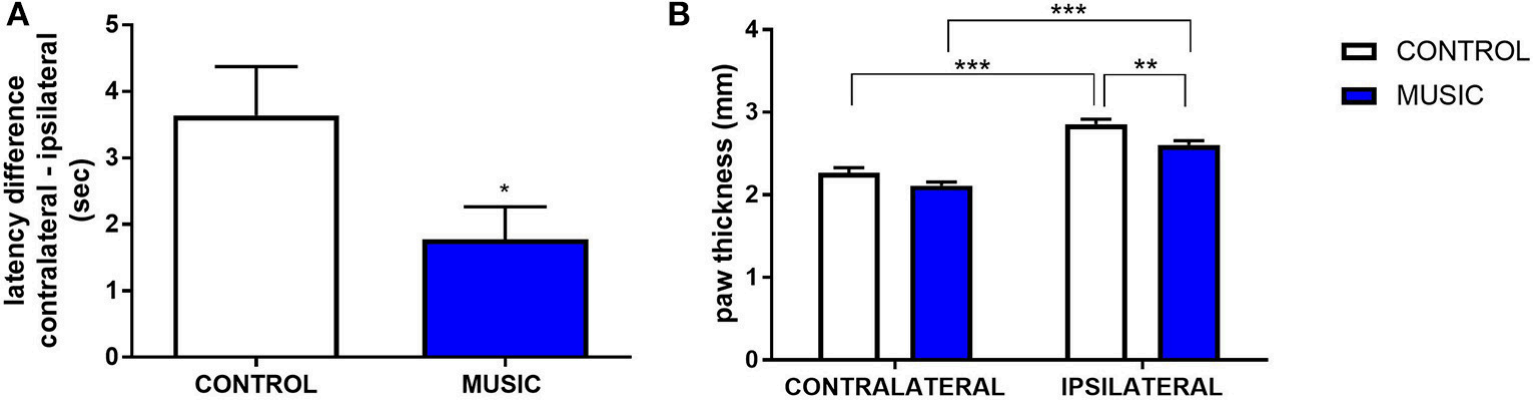
Analgesic effects of music-based intervention in the carrageenan model of inflammatory pain in CF-1 mice. **(A)**—effects of 21-day exposure to the Mozart playlist (MUSIC–blue; CONTROL–white) on paw withdrawal latency difference. Latency differences (contralateral PWL–ipsilateral PWL) show that hyperalgesia was observed in animals exposed to control conditions and music-exposed mice show a reduced latency difference.^*^P < 0.05. **(B)**—Effects of music-based intervention on paw thickness (edema) following carrageenan administration in CF-1 mice. Edema was observed following carrageenan in animals exposed to control conditions whereas music-exposed animals show a diminished edema (MUSIC–blue; CONTROL–white). ^**^P < 0.01, ^***^P < 0.001. *N* = 5–8 per group. Data were analyzed using either a *t*-test **(A)** or a two-way ANOVA followed by a Bonferroni *post-hoc* test **(B)**.

In CD-1 mice, we first determined whether hyperalgesia following intraplantar carrageenan would be reduced in music-exposed animals. CD-1 mice (*N* = 5–8 per group) were evaluated for thermal hyperalgesia following carrageenan administration and, as shown Figure 1A and Table 2, there were no significant differences in PWL between music-treated and control groups. We then selected several analgesic compounds, including approved analgesic drugs or novel analgesic drug candidates, to be evaluated in control and music-exposed mice. Moderate or sub-therapeutic doses of each compound (data not shown) were therefore evaluated in combination with music treatment. Mice receiving an acute dose of ibuprofen showed a significant reduction in hyperalgesia when treated in combination with music, as demonstrated by an increase in post-carrageenan PWL in ibuprofen + music-exposed mice, whereas other compounds did not significantly reduce this hyperalgesic response to carrageenan (Figure 1B and Table 2). Plantar edema was also evaluated in all carrageenan-treated mice and it was observed that edema was reduced when music exposure was paired with specific compounds. As illustrated in Figure 2 and Table 2, we observed a reduction in post-carrageenan paw thickness in music-exposed mice co-treated with the galanin analog NAX 5055 or cannabidiol. This anti-edema effect was not observed with other drugs tested. In addition to testing in CD-1 mice, CF-1 mice were also evaluated in the carrageenan assay following exposure to music. The CF-1 animal strain was used for evaluation of anti-seizure effects of music exposure and therefore this animal strain was also included in analgesic testing in the carrageenan assay. In CF-1 animals, a moderate analgesic effect of music was observed, as the difference score of PWL (contralateral latency—ipsilateral latency) was reduced in music-exposed mice (Figure 3A). Furthermore, there was a reduction in carrageenan-induced edema in music-treated mice (Figure 3B).

**Table 2:** Latency and edema differences (vs. contralateral) for both the control (ambient noise) and music-exposed CD-1 mice treated with various analgesic compounds and evaluated in the carrageenan model of inflammatory pain.

**Compound**	**Latency difference (sec)**		**% of Contralateral paw thickness**	
	**Control**	**Music**	**Control**	**Music**
Vehicle	3.68 ± 0.68	2.79 ± 0.69	149.9 ± 5.6	137.6 ± 7.5
Galanin analog NAX 5055, 4 mg/kg	2.91 ± 0.61	1.65 ± 0.60	130.7 ± 2.5	119.5 ± 3.6^*^
Levetiracetam, 400 mg/kg	2.30 ± 0.58	2.07 ± 0.50	158.7 ± 5.8	137.9 ± 10.50
Cannabidiol, 100 mg/kg	2.18 ± 0.50	1.39 ± 0.64	167.0 ± 9.34	133.2 ± 1.83^**^
Ibuprofen, 25 mg/kg	2.57 ± 0.90	0.18 ± 0.48^*^	143.0 ± 4.60	135.9 ± 1.6

Means ± standard error, N = 5–8 per group.

* P < 0.05 compared to control.

** *P < 0.01 compared to control. Data were analyzed by t-test (music-exposed vs. control for each treatment arm)*.

**Figure 4:**
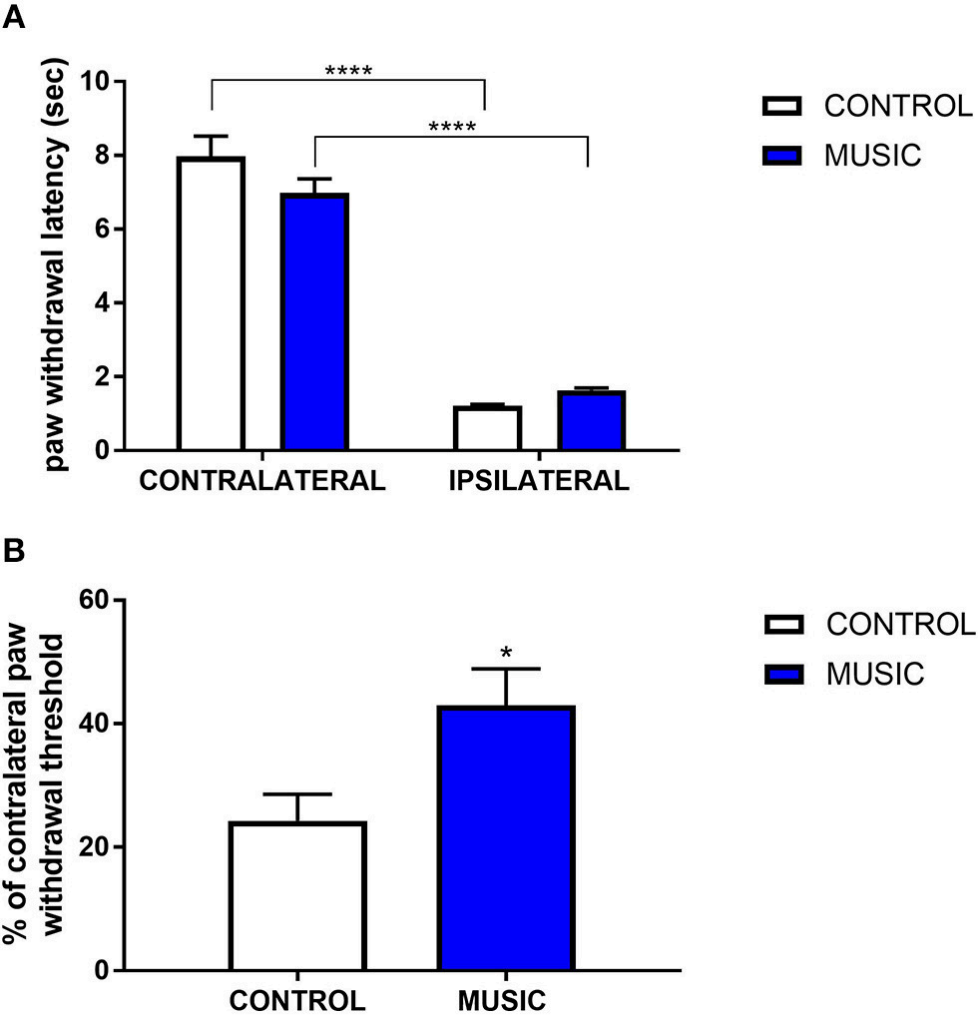
Effects of music intervention in plantar incision model of surgical pain in CD-1 mice. **(A)**—Paw withdrawal latency (PWL; sec) following thermal stimulation in mice following plantar incision. Incised paws (ipsilateral) show a greatly diminished PWL as compared to non-incised (contralateral) paws. ^****^P < 0.0001. **(B)**—Paw withdrawal threshold to mechanical stimulation following plantar incision. Thresholds in ipsilateral paws are shown as a percentage (%) of the contralateral paw withdrawal threshold. ^*^P < 0.05. *N* = 13–15. Data were analyzed using a two-way ANOVA followed by a Bonferroni *post-hoc* test **(A)** or a *t*-test **(B)**.

In addition to the inflammatory model of pain, we evaluated thermal pain and mechanical allodynia in music-exposed mice in a model of post-surgical pain. Incisional pain results from a combination of innate factors responding to this acute tissue injury, and involves both peripheral and central pain pathways. There were no significant differences in PWL to thermal stimulus between the music and control groups (Figure 4A), whereas we observed significant increase in the paw withdrawal threshold (incised (ipsilateral) paw normalized to contralateral paw) following mechanical (von Frey) stimulation (Figure 4B). The variance in response following plantar incision suggests a greater benefit of music exposure in mechanical stimulation rather than thermal stimulation. Additional studies are needed to further explore the mechanisms whereby music preferentially affects mechanical allodynia in this model.

### Epilepsy Studies

**Figure 5:**
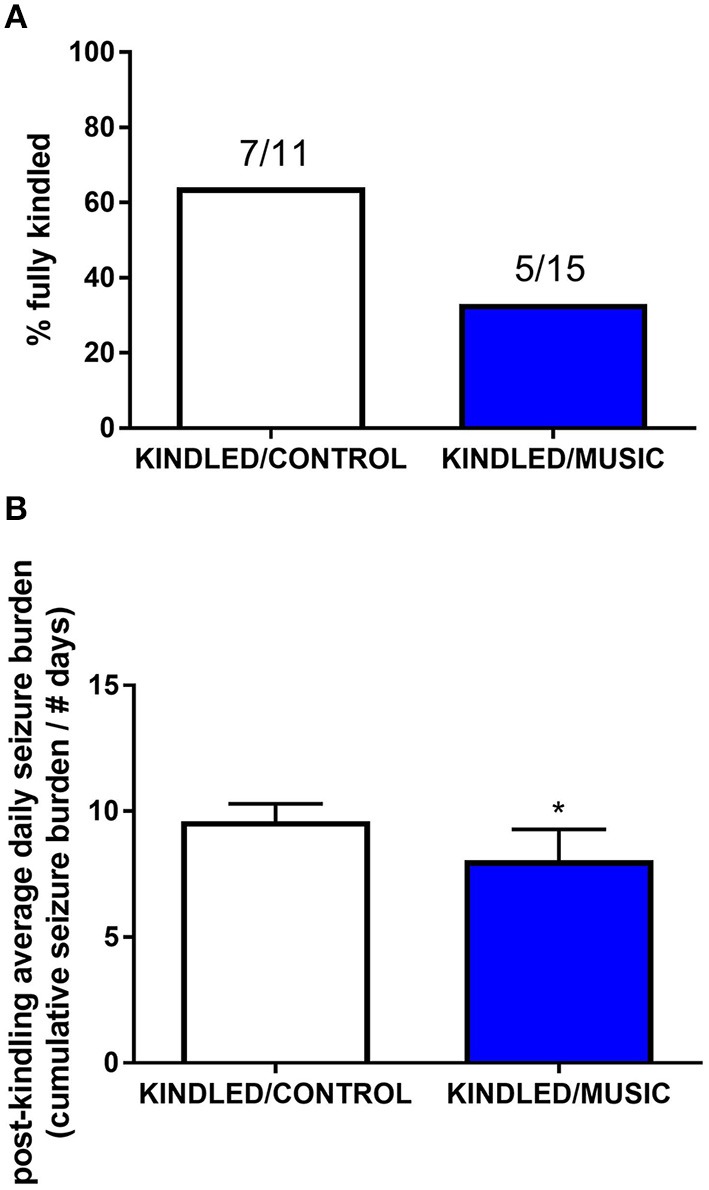
Antiseizure effects of music-based intervention in the corneal kindling model of epilepsy in CF-1 mice. **(A)**—The percentage of fully kindled animals, as well as the number of fully kindled animals (number reaching fully kindled status / N; e.g., 7/11 and 5/15 for CONTROL- (white) and MUSIC (blue)-treated groups, respectively), is shown for each treatment group. **(B)**—The cumulative seizure burden (sum of all Racine scores for each stimulation) for all animals following achievement of fully kindled status. Mice exposed to music showed a lower post-kindling seizure burden. ^*^P < 0.05 compared to control kindled. Data were compared by a Fisher's exact test **(A)** or a *t*-test **(B)**.

**Figure 6:**
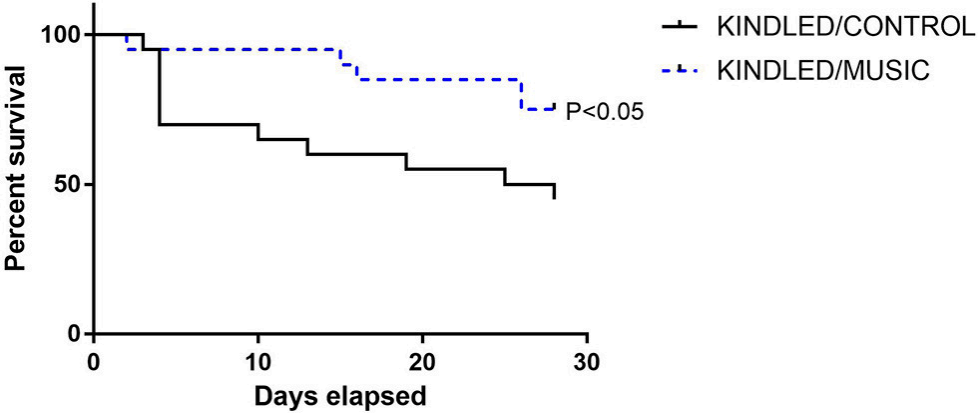
Reduced mortality in animals exposed to daily music during kindling development. A survival analysis was conducted for all animals (*N* = 20 per group at study start) during kindling acquisition in CF-1 mice. While nearly 50% of control kindled animals die by the end of kindling acquisition, a significantly lower portion of mice in the music (music + kindling) died during this period. Groups were compared by Log-rank (Mantel-Cox) and Gehan-Breslow-Wilcoxon tests.

Previous studies suggested that exposure to the Mozart music had antiseizure effects in rat model of absence seizures (34), and can reduce cognitive impairment in rat model of status epilepticus (54). We tested effects of the Mozart playlist on seizures in the corneal kindling model of epilepsy in CF-1 mice. Exposure to music did not affect the rate of kindling. There were no significant differences between music-exposed and control mice in acquisition of a fully kindled state (data not shown). The mean number of stimulations required for the first seizure to be observed was 4.2 ± 0.7 and 4.5 ± 0.5 for the control and music-exposed groups, respectively. Similarly, there were no differences in the number of stimulations to reach the first generalized seizure (11.1 ± 2.3 and 11.1 ± 1.2 for control and music-exposed groups, respectively). It also took a similar number of stimulations to achieve a fully kindled status (33.6 ± 2.9 and 37.5 ± 2.6 for control and music-exposed groups, respectively). Interestingly, as summarized in Figure 5, though there were no significant differences in the number of animals that reached a fully kindled state (Figure 5A), once kindling was established there was a significant reduction of seizure burden (cumulative score of behavioral seizures) in music-exposed mice (Figure 5B). Furthermore, music-exposed CF-1 mice had a significantly higher survival rate (Figure 6).

**Figure 7:**
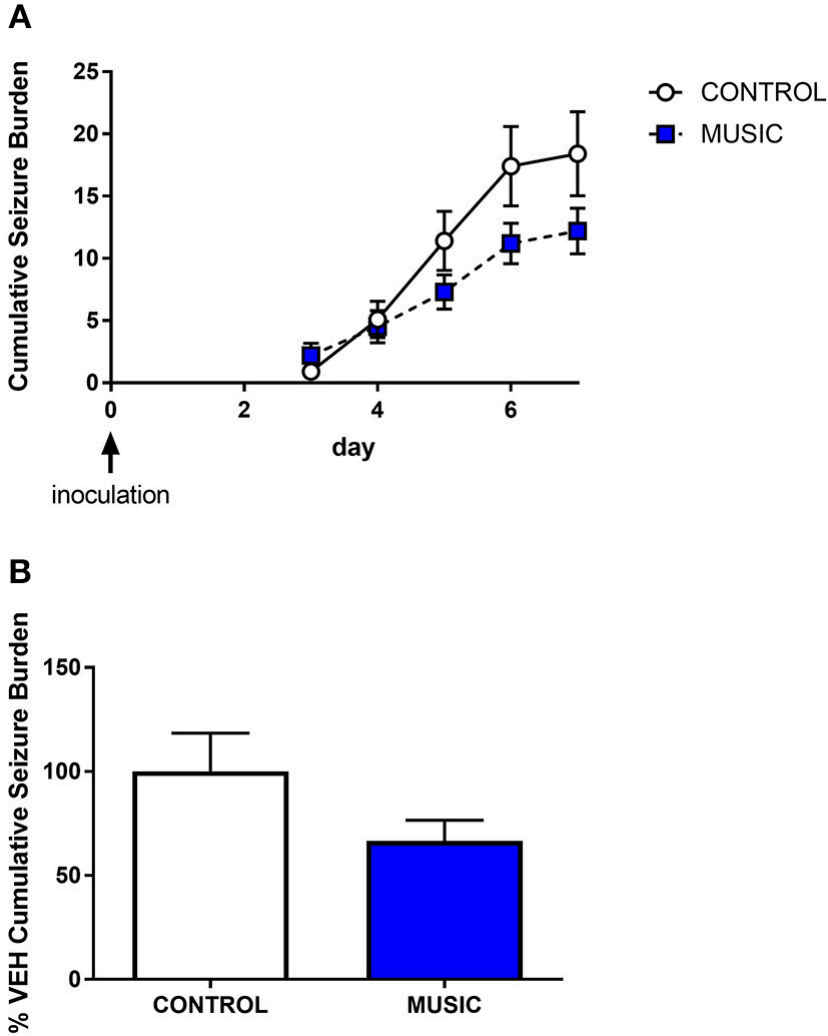
Evaluation of the effects of music treatment on handling-induced seizures in the TMEV model of acquired epilepsy. Groups of mice (*N* = 10) were exposed to either control conditions or music for a 3-week period prior to inoculation with TMEV. Daily handling sessions occurred during day 3—day 7 post-inoculation wherein seizures were scored (Racine scale). The cumulative seizure burden during this time is shown in **(A)**. The final cumulative seizure burden, expressed as a percentage of control-treated animals, is shown in **(B)**. Groups were compared by a Mann-Whitney *U*-test.

In order to evaluate the potential effects of music exposure in an inflammatory model of epilepsy, we also tested control and music-treated mice for the presence of handling-induced seizures in the TMEV model of epilepsy. Prior exposure to music did not have an effect on the presence of handling-induced seizures in this model. Therefore, following intracortical administration of TMEV, which produces a period (5–10 days) of increased seizure susceptibility, both music-exposed and control mice show similar handling-induced seizure burden (see Figure 7). Although there were no significant differences in seizure burden observed in the TMEV assay, it is noteworthy that music exposure was stopped on the evening prior to inoculation. Therefore, given the potential for music exposure to mediate central inflammatory responses, additional work is needed in this model that includes music exposure throughout the acute infection period.

## Discussion

There is an increasing interest in non-pharmacological therapies for the treatment of a number of disease states, including chronic pain management. For example, the American College of Physicians updated clinical guidelines recommending physical exercise and yoga as the first line therapy for lower back pain (75). While there are many animal studies on exercise-induced analgesia (76, 77), analgesic properties of music have been sparsely investigated in animal models (43) in contrast to growing clinical evidence (22, 24–26, 78–82). To the best of our knowledge, this is the first study in animal models of pain illustrating how exposure to music can enhance the antinociceptive activities of analgesic drugs. Given gastrointestinal and cardiovascular toxicity of NSAIDs, and liabilities of opioid-based analgesics, our current work supports further investigation of combining music-based interventions with analgesic compounds to develop novel therapies for pain. Our preliminary results that music treatment concurrently reduced pain and paw edema in the inflammatory model of pain warrants more studies in arthritis-related animal models.

The antiseizure effects of the Mozart music in mice models of epilepsy confirmed previously published observations in rats (34). Given the clinical evidence supporting antiseizure effects of Mozart music in patients with epilepsy (28, 33, 35–42, 83), and preclinical evidence that the Mozart music can upregulate expression of BDNF in the rat hippocampus and reduce cognitive impairment in status epilepticus model in rats (54, 84, 85), our findings warrant further investigation in other models of epilepsy and epileptogenesis. This work also supports research on the Mozart music in canine epilepsy (86). The decrease in the kindling-related mortality in CF-1 strain of mice (but not CD-1) was significant and unexpected, although the Mozart music as a preventive therapy for SUDEP was previously suggested (87). One possible mechanism by which Mozart music may improve mortality is via cardiovascular effects (33, 55, 83, 88), also relevant in the kindling experiments (89). Our current results encourage more in-depth studies in SUDEP animal models.

In these studies, we utilized multiple strains of mice to evaluate the effect of music exposure on evoked pain and seizure acquisition. CD-1 mice are commonly used for pharmacologic and behavioral testing and therefore this strain was used for analgesia assays. However, we observed that this strain of mice does not survive kindling stimulation and therefore was not evaluated in this model (data not shown). By contrast, CF-1 mice are routinely used in the kindling model and demonstrate a robust and reproducible kindling acquisition rate (68, 90). The reduction in mortality and post-kindling seizure burden in the kindling model suggests the potential for music-mediated disease burden and validates use of this strain for additional testing. Therefore, we also evaluated this strain following music exposure in pain models. Beneficial effects of music in the kindling and pain models is suggestive of a potential anti-inflammatory effect. Therefore, we also sought to evaluate anti-seizure effects of music exposure in the TMEV model of epilepsy. This model requires C57Bl/6J mice and therefore this strain was used (91, 92).

We acknowledge many limitations of this present study which was focused on surveying music effects in mouse pain and epilepsy models, rather than in-depth mechanistic studies. Daily exposure to the Mozart music was limited to only 3 weeks prior to most tests, and this duration was based on previous studies in rats (34). However, it is unclear if longer durations could have more pronounced effects given that we observed variations in effects of music depending on a strain of mice (CD-1 vs. CF1). In pilot experiments, we did not observe significant differences in seizure thresholds in the 6 Hz, MES and minimal clonic seizure models in the CD-1 strain, and no additional antiseizure effects were observed in the presence of levetiracetam and clobazam (data not shown). Another limitation was the use of ambient noise for the control group instead of a “negative” control (e.g., playing another type of music or white noise). However, a negative control (retrograde inversion of Mozart's K.448) was previously used to show that specific musical structures in Mozart's K.448, such as rhythm, could account for an increased expression of BDNF in the rat hippocampus (85). Also specific physiological effects of the Mozart's music (as compared to “other” music) were reported elsewhere (55, 93), while there were no differences in expression of BDNF between control (no sound) and the white noise as compared to music intervention in mice (44). Further optimization of exposure to music (daily exposure, total duration, volume, compilation of specific musical compositions) is essential before more conclusions can be drawn regarding what types of epileptic seizures and what types of pain are most sensitive to the music-based intervention.

### Mechanisms of Analgesic and Antiseizure Effects of Music

Functional effects of music on the nervous system have been extensively studied (56, 94–99). Music is complex by nature thus creating additional challenges when dissecting a mechanism of action for its analgesic and antiseizure properties. Herein, we hypothesize that neuroactive effects of music may involve: (1) the brain neuroplasticity through upregulation of BDNF (84, 85), (2) modulation of the parasympathetic tone (28, 33, 83), and (3) possible contributions from the dopaminergic system (4, 28, 100) and opioid receptors (101). Since music was shown to decrease stress hormone cortisol (94, 102, 103), an additional target for music-evoked antiseizure and analgesic activities can be modulation of the HPA axis (103–108) and proinflammatory cytokines such as IL-6 (94, 95, 109–112). However, it is important to note that these potential mechanisms were not evaluated in the current study and further investigation is required to test this underlying hypothesis.

Our experiments showed more profound outcomes of music in pain models as compared to those in epilepsy models. These observations can be accounted for by numerous factors. For example, our study protocol using a 3-week exposure to music could favor responses associated with sub-chronic modulation of the HPA axis and a reduction in post-insult cytokine release. Further, anti-inflammatory effects of music exposure may have multiple sites of action, including central (spinal) and peripheral (dorsal root ganglion and nociceptor-mediated) effects. If neuroplasticity-based changes in excitatory and inhibitory pathways in the brain play a significant role in music-evoked antiseizure effects, then longer-term exposure to music may produce more outcomes in epilepsy animal models. Noteworthy, Mozart music was shown to yield time-dependent increase in the BDNF expression in the rat hippocampus with the highest levels after 98 days (85). Due to limited data, it is currently too speculative to infer differences in mechanisms involved in music-mediated analgesia and antiseizure effects.

**Figure 8:**
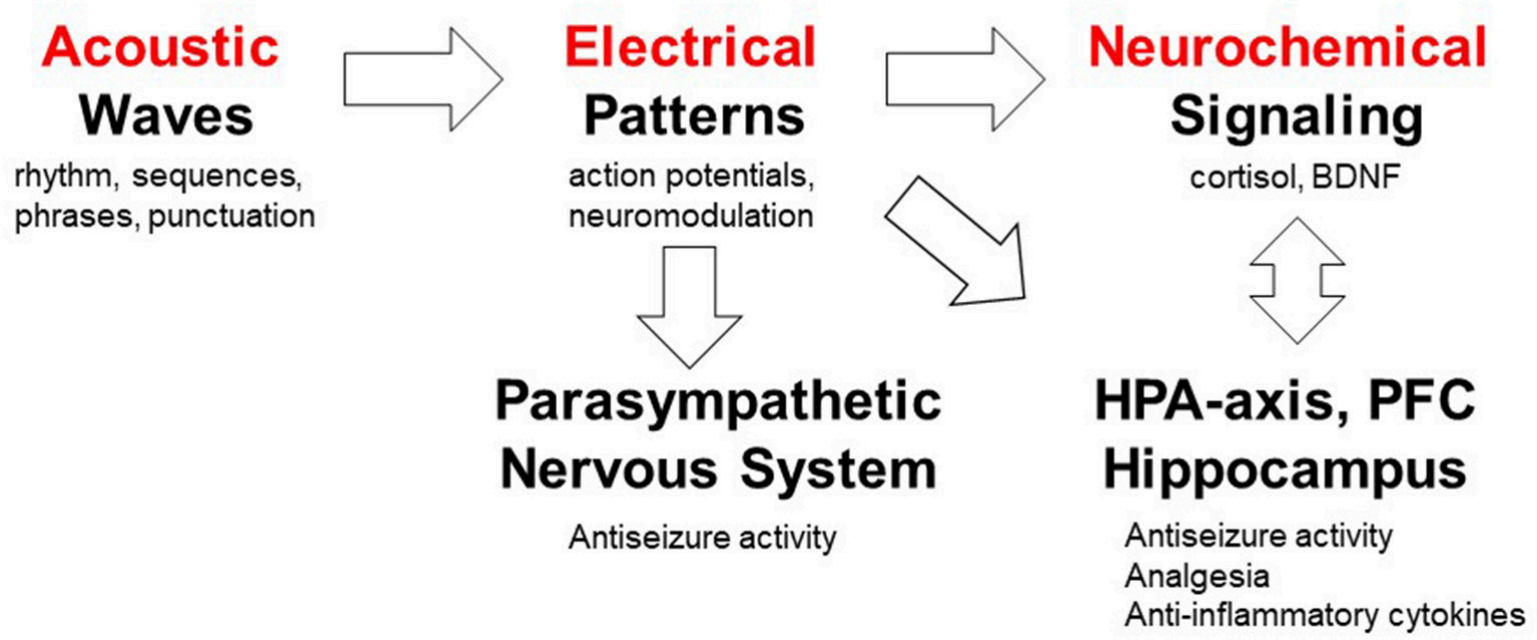
Working model of mechanisms by which musical compositions can exert their analgesic and anticonvulsant activities. This model serves as a platform for testing a number of specific hypotheses; not addressed in the present investigation. The auditory system processes acoustic waves with specific rhythm, sequences, phrases and punctuation which generate action potentials in the nervous system. The role of specific musical structures (rhythm and pitch) in K.448 was studied in rodents and humans (85), whereas high periodicity was proposed to account for the antiseizure effects (73). Musical tempo modulate emotions (113) which can in turn affect pain processing (114, 115). Exposure to K.448 was shown to activate the parasympathetic nervous system (33). Music was shown to modulate the hypothalamic-pituitary-adrenal (HPA) axis, decrease stress hormone cortisol and increase expression of BDNF in the hippocampus (44, 57, 85, 94, 95, 103). The roles of prefrontal cortex (PFC) in pain processing (116) and music processing (97) have been studied. Further studies are required to test mechanism(s) of action of music-enhanced analgesia and antiseizure activities.

Interestingly, the Mozart music was shown to be “active” in both humans and rodents, suggesting that a more universal model may explain its “medicinal” properties. As illustrated in Figure 8, our long term goal is to delineate how specific musical structures (auditory stimulation) can be translated into electrical patterns in the brain and the peripheral nervous system, and subsequently into neurochemical signaling pathways leading to reduction of seizures or antinociception. From a translational perspective, interspecies differences in processing sound must be taken into account. For example, mice hearing extends into the ultrasonic frequencies and ranges from 1 to ca. 100 kHz; by contrast, human hearing ranges from 20 to 20 kHz. In addition, “Hearing is most sensitive for humans at frequencies of approximately 1 to 4 kHz and approximately 16 kHz for mice. Misunderstanding of the differences in sensitivity to sound of different frequencies across species could lead to the incorrect assumption that if humans can hear a sound, mice can hear it as well.” (117). Studies suggest that rodents may evolved the ability to process musical rhythms via midbrain (118). Since the antiseizure effects of the Mozart music were also observed in people with epilepsy when music was delivered during sleep (42), we hypothesize that rhythmic and melodic structures may exert diverse and overlapping effects when processed by the brainstem, midbrain and forebrain.

Likely musical elements in the Mozart K.448 that can in part account for the observed effects are rhythm and tempo, phrase structure and punctuation (or cadence). Indeed, this may be due to the fact that there is a connection between a basic periodic pulse in perception and physical and physiological activities. “Many activities, such as sucking in newborn infants, rocking, walking, and beating of the heart, occur with periods of approximately 500 ms to 1 s.” (119) The notion of periodicity is important because the Mozart's music is replete with periodic repetition. Next, another of Mozart's pieces, the piano sonata in C Major, K.545 has also been found to have similar effects. Accordingly, a comparative analysis of the first movement of each piece yielded the following features that align with periodic repetition: rhythm (via use of a rhythm schemata), phrase length (specifically the Classical ideal of the four-bar phrase) and the related phenomenon of punctuation, and melodic sequences. In regards to rhythm, the first movement of both pieces have a continuous flow of sixteen notes. However, both pieces have objective rhythmization (in the case of K.545—for instance—there is an eighth note on each downbeat throughout the transition). The four-bar phrase and melodic sequence demonstrate periodicity at a higher level (i.e., there is more content). It seems likely that the periodic structure present in both pieces may exert their physiological effects, but more experiments are needed in order to support this hypothesis.

### Translational Implications of Studying Enriched Environment

**Figure 9:**
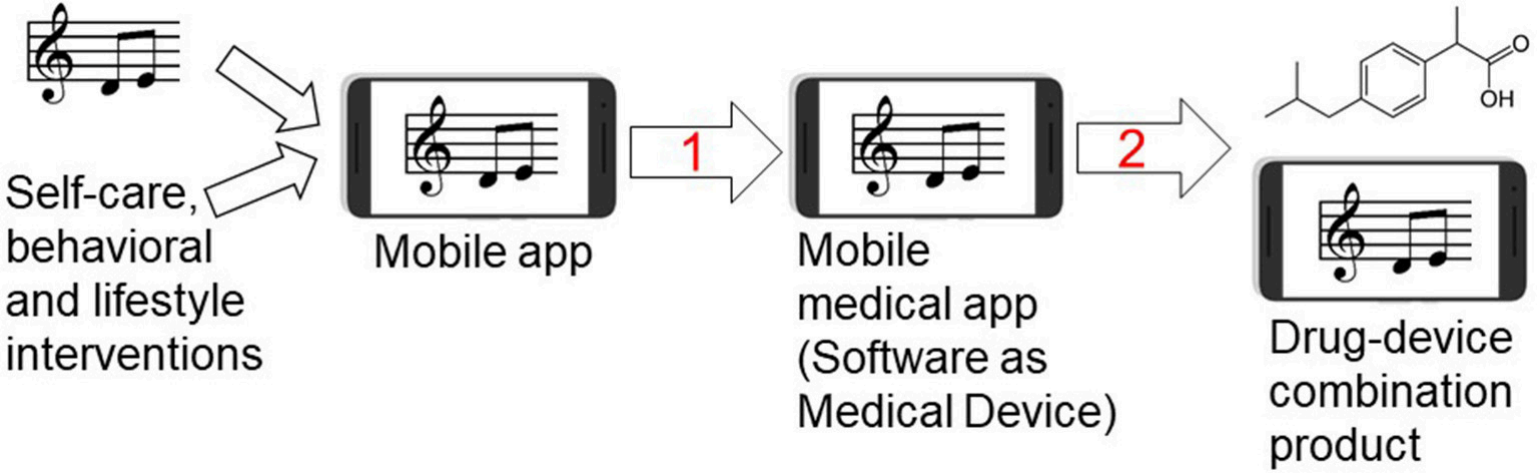
Developing music-based and behavioral interventions and their combinations with pharmaceutical drugs using digital therapeutics strategy. Streaming of patient-preferred music can be combined with disease self-management and behavioral therapy yielding a mobile app for non-pharmacological interventions. Step 1: Once the mobile app is clinically tested for efficacy in pivotal randomized controlled trial (RCT) and receives the regulatory approval or clearance, it becomes a mobile medical app (software as a medical device). Step 2: testing clinical efficacy of combining a pharmaceutical drug (ibuprofen structure is shown as an example of an analgesic drug) with the mobile medical app can lead to premarket application for the regulatory approval of drug-device combination product in which the mobile medical app is a medical device.

Digital therapeutics including mobile medical apps and video games have been developed and already received the regulatory approval for the treatment of diabetes, addiction, stroke and traumatic brain injury (Table S1). For example, following a pivotal clinical study, the music-based video game, MusicGlove, received regulatory clearance by the Food and Drug Administration as a stroke therapy (120, 121). Positive data from clinical studies of digital technologies for the treatment of pain suggest an emergence of “digital analgesics” (11, 13, 14). Using digital therapeutics to deliver non-pharmacological interventions such as music and/or physical exercise creates new opportunities for combining these modalities with pharmacotherapies and clinically-validated natural products (4–6). While development of digital therapeutics does not require preclinical testing (in contrast to regulatory requirements for investigational new drug (IND) enabling studies), translational implications of our study include: (1) using EE as a preclinical surrogate for testing combinations of non-pharmacological modalities for the treatment of pain and other chronic diseases, and (2) preclinical testing and development of drugs in the presence of non-pharmacological interventions (e.g. music, physical exercise, nutritional therapy, EE) eventually leading to novel drug-device combination products for the treatment of pain, epilepsy, depression, cancer and other chronic diseases. Many failures of investigational new drugs to reach primary end points in pivotal clinical studies underscore opportunities for co-development of digital therapeutics as innovative combination therapies. Given parallel advances in developing new analgesics (122) and mobile apps for pain (15), drug-device combination products offer seamless integration and delivery of two modes of action simultaneously. This paradigm is further illustrated in Table 3 and Figure 9.

**Table 3:** Feasibility of using preclinical studies to support development of drug-device combinations of digital therapeutics with respective pharmacotherapies for chronic diseases.

**Indication**	**Non-pharmacological intervention**			**Pharmacotherapy**
	**Modality**	**Preclinical study**	**Digital technology**	
Pain	Music	This work Cancer pain model (43)	Mobile app delivering music (16)	Analgesics
Pain	Physical exercise	Exercise-induced analgesia (123, 124)	Mobile app delivering exercise (125)	Analgesics
Epilepsy	Music	This work Absence seizures model (34)	Mobile app delivering music and self-care (6)	Antiseizure drugs
Depression	Cognitive stimulation	Enriched environment (61)	Mobile app delivering behavioral intervention (126)	Antidepressants
Cancer	Physical exercise	Gentle stretches (127) Enriched environment (128)	Exercise-empowerment video game (129)	Anticancer drugs
Multiple sclerosis	Physical exercise	Enriched environment (130)	Mobile health exercise app (131)	Immunomodulators

Table 3 demonstrates how employing EE can be useful for testing combinations of non-pharmacological interventions and pharmacological compounds, thus potentially improving their efficacy and potency. For pain, preclinical and clinical studies suggest that EE and behavioral interventions exert analgesic effects (45, 132, 133). Music has been shown to produce analgesia in humans (25–27, 80, 82, 134) and in rats (43). Recent studies also suggest analgesic activities of exposure to light in both humans and rats (135, 136). The antinociceptive effects of the green LED light were associated with down-regulation of N-type calcium channels in dorsal root ganglion neurons, as well as were reversed by naloxone, thus also implicating the opioid-based analgesic mechanism (136). Herein, we propose that studying combinations of multiple non-pharmacological modalities can lead to non-invasive and non-addictive treatments of pain (“digital analgesics”) which can also result in lowering effective doses of analgesics and improving pain relief. To the best of our knowledge, there are no published results on combining analgesics with EE, except those studies indirectly suggesting beneficial interactions between analgesic neuropeptide galanin and physical exercise (137–139).

As illustrated in Figure 9, behavioral therapies, music and disease self-management can be combined with specific prescription medications using drug-device combination product regulatory mechanism, perhaps leading to reducing adverse effects and improving patient engagement in therapies (4). This drug-device combination strategy has apparent benefits to treat pain or epilepsy because digital therapeutics can simultaneously target depression as a comorbidity. Since antidepressants and psychotherapies have comparably low remission rates (30–40%) in patients with depression (140), delivering depression-related digital content (e.g., physical exercise and music) may help to ameliorate depressive symptoms (20, 23, 126, 141–143). From preclinical development perspectives, it is worth mentioning that EE was shown to reduce seizures in temporal lobe epilepsy model in rats (144) and depressive symptoms after seizures (49). Our study also has implications for other chronic medical conditions including cancer. Music and EE-based interventions may serve as preclinical surrogate for developing adjunct digital therapeutics for cancer patients (43, 58, 127), since music can lower cancer-treatment biomarker IL-6 (94, 112, 145) while EE and physical exercise can reduce tumor size and increase lifespan in cancer animal models (127, 128, 146). Other clinical opportunities to combine non-pharmacological modalities with pharmaceutical drugs and biologics include Parkinson's and Alzheimer's diseases (21), asthma (147), arthritis (148) and affective disorders (143, 149).

From a drug development perspective, preclinical studies of non-pharmacological intervention (e.g., music, physical exercise) to improve potency, efficacy and therapy outcomes of IND candidates can be translated into randomized controlled trails in which IND candidates are clinically tested in conjunction with digital therapeutic delivering the same type of non-pharmacological intervention. Such innovative approaches to developing drug-device combination therapies may be further incentivized by unique ability of mobile apps to harness GPS data for just-in-time adaptive interventions adjusted for weather, air quality, or even seasonal changes (150–154). The opportunity to tailor digital content based on forecast and current atmospheric conditions was mentioned as a qualitative feedback from a patient when testing a mobile app for pain self-management (155). Taken together, delivering non-pharmacological interventions by mobile technologies offers innovative means to improve therapy outcomes for pain, epilepsy and other chronic disorders.

## Conclusion

Our current study suggest that music-enhanced analgesia may lead to novel combination therapies comprising music and analgesic drugs, whereas similar combinations for the treatment of epileptic seizures need to be further investigated. Music-based intervention can be integrated with other non-pharmacological modalities and delivered as digital therapeutics for pain, epilepsy, depression and other chronic medical conditions. This work opens new opportunities for employing music and EE as surrogate for discovering synergistic effects between non-pharmacological and pharmacological interventions and leading to innovative drug-device combination therapies for chronic disorders.

## Data Availability

All datasets generated for this study are included in the manuscript and/or the supplementary files.

## Author Contributions

CM, MH, MDS, HSW, and GB designed experiments. MH and GG designed music based intervention. MH, GG, MC, and EW analyzed music. CM, MH, AK, TU, FV, and GB performed experiments. CM, MH, MDS, HSW, and GB analyzed and discussed results. CM, MH, AK, MC, EW, and GB wrote the manuscript. CM, MDS, HSW, and GB edited the manuscript.

### Conflict of Interest Statement

GB is a co-founder, officer and the board member in Epicadence, Public Benefit Corporation, focused on development of mobile software for people with epilepsy. GB is a co-inventor on patent-pending “Multimodal Platform for Treating Epilepsy” licensed to Epicadence PBC. CM, GB, and HSW are co-inventors on patented technology related to engineering galanin analogs including NAX-5055 and their use as analgesics and anticonvulsant drugs. The patents are owned by the University of Utah. GG is a founder and owner of Greatful Living Productions. The remaining authors declare that the research was conducted in the absence of any commercial or financial relationships that could be construed as a potential conflict of interest.

## Abbreviations

BDNF: brain-derived neurotrophic factor
CBD: cannabidiol
EE: enriched environment
HPA: hypothalamic-pituitary-adrenal
IND: investigational new drug
KA: kainic acid
LEV: levetiracetam
PBS: phosphate-buffered saline
PFC: prefrontal cortex
PWL: paw withdrawal latency
SaMD: software as medical device
SUDEP: Sudden Unexpected Death in Epilepsy
TMEV: Theiler's murine encephalomyelitis virus
TPE: time-to-peak effect.
