# Supplementary material for: Music-Enhanced Analgesia and Antiseizure Activities in Animal Models of Pain and Epilepsy: Toward Preclinical Studies Supporting Development of Digital Therapeutics and Their Combinations With Pharmaceutical Drugs
Source: Front Neurol. 2019 Mar 27;10:277. doi: 10.3389/fneur.2019.00277 (PMC6446215; doi:10.3389/fneur.2019.00277)
Supplement: Supplementary file 1 [file Table_1.pdf]

Supplementary Material

Music-enhanced Analgesia and Antiseizure Activities in Animal Models of Pain and Epilepsy: Towards Preclinical Studies Supporting Development of Digital Therapeutics and their Combinations with Pharmaceutical Drugs

Cameron S Metcalf, Merodean Huntsman, Gerry Garcia, Adam K. Kochanski, Michael Chikinda, Eugene Watanabe, Tristan Underwood, Fabiola Vanegas, Misty D Smith, H Steve White, Grzegorz Bulaj

Table S1. Examples of digital interventions approved or cleared by the US Food and Drug Administration as "software as a medical device", and intended to treat specific chronic medical conditions.

Name and type of product	Indication	Website of the company
BlueStar, mobile app	Diabetes type 2	https://www.welldoc.com/
Music Glove, video game	Stroke, Traumatic brain injury	https://www.flintrehab.com/
Jintronix. video game	Stroke, Traumatic brain injury	http://www.jintronix.com/
reSET, mobile app	Substance use disorder, non-opioid	https://peartherapeutics.com/
reSET-O, mobile app	Opioid use disorder	https://peartherapeutics.com/

Note: As of resubmission of this manuscript, digital therapeutic AKL-T01 for the treatment of ADHD (video game Evo developed by Akili Interactive) is under regulatory review from the FDA. Reference: https://www.akiliinteractive.com/
